# A Contextual Fire Detection Algorithm for Simulated HJ-1B Imagery

**DOI:** 10.3390/s90200961

**Published:** 2009-02-13

**Authors:** Yonggang Qian, Guangjian Yan, Sibo Duan, Xiangsheng Kong

**Affiliations:** 1 State Key Laboratory of Remote Sensing Science, School of Geography, State Key Laboratory of Remote Sensing Science, Beijing Normal University, 100875, Beijing, P.R. China; E-Mail: qianyg2000@163.com (Y.Q); 2 Institute of Advanced Computing and Digital Engineering, Shenzhen Institute of Advanced Technology,Chinese Academy of Sciences, 518054, Shenzhen, P.R. China; 3 The Geography and Planning College of Ludong University, 264025, Yantai, P.R. China

**Keywords:** Fire detection, simulated HJ-1B imagery, contextual algorithm

## Abstract

The HJ-1B satellite, which was launched on September 6, 2008, is one of the small ones placed in the constellation for disaster prediction and monitoring. HJ-1B imagery was simulated in this paper, which contains fires of various sizes and temperatures in a wide range of terrestrial biomes and climates, including RED, NIR, MIR and TIR channels. Based on the MODIS version 4 contextual algorithm and the characteristics of HJ-1B sensor, a contextual fire detection algorithm was proposed and tested using simulated HJ-1B data. It was evaluated by the probability of fire detection and false alarm as functions of fire temperature and fire area. Results indicate that when the simulated fire area is larger than 45 m^2^ and the simulated fire temperature is larger than 800 K, the algorithm has a higher probability of detection. But if the simulated fire area is smaller than 10 m^2^, only when the simulated fire temperature is larger than 900 K, may the fire be detected. For fire areas about 100 m^2^, the proposed algorithm has a higher detection probability than that of the MODIS product. Finally, the omission and commission error were evaluated which are important factors to affect the performance of this algorithm. It has been demonstrated that HJ-1B satellite data are much sensitive to smaller and cooler fires than MODIS or AVHRR data and the improved capabilities of HJ-1B data will offer a fine opportunity for the fire detection.

## 1. Introduction

Biomass burning has tremendous impact on the Earth's ecosystems and climate, as it drastically alters the landscape and biological structure, and emits large amounts of greenhouse gases and aerosol particles [1]. Smoke aerosols may interact with cloud droplets and considerably change the earth's radiation budget [2-3]. It is important to recognize that changes in climate can affect changes in fire regime, which is a combination of the type of fire which occurs in a given region, the frequency at which fires occur, and the seasonality of burning. Consequently, there is the potential for substantial feedback between fire and the environment.

Global fire detection is thus a very important issue for humans and environment. Whether fire detection can be effective, timely and accurate or not, will correlate directly with the security of human life and the degree of destruction of the environment. Remote sensing is able to monitor the fire activity over a wide range. An increasing number of programs have been established with the goal of obtaining information about and determining the fire regime by satellite, e.g., the International Geosphere-Biosphere Program, Data and Information System's (IGBP-DIS) Global Fire Product initiative [4], the ASTR World Fire Atlas [5-6] and the MODerate Resolution Imaging Spectro-radiometer (MODIS) Fire Product [7].

Different sensors have both advantages and limitations for global and regional fire detection. Geostationary satellites can acquire data every 15 to 30 minutes over a given area, but any given one of them may only cover a portion of the Earth's surface. Further, they generally provide very low spatial resolution (4 Km pixel size or larger). On the other hand, current satellite sensors with high spatial resolution such as the Landsat Thematic Mapper (TM), which has 60 m resolution in the thermal band, often cannot cover the global area quickly, and only provide a 16 days revisit period for most parts of the Earth.

The Advanced Very High Resolution Radiometer (NOAA-AVHRR) has a long history in fire detection. It offers a spatial resolution of 1 km and covers most of the Earth's surface every day. However, its Mid-infrared (MIR) channel has a low saturation of 320 k, which limits the use for fire detection [2, 8]. MODIS on Terra and Aqua can cover most of the world at least four times a day (twice during the day and twice during the night). Meanwhile, MODIS enhances the saturation brightness temperature to 500 K and 340 K for the MIR and TIR channels, respectively [7, 9-10]. MODIS is expected to play an important role in global fire detection and monitoring, but its nadir spatial resolution of 1 km may not be fully sufficient for the early detection of small fires, resolution of individual fire fronts and fire intensity estimation [7]. For smaller fires, MODIS' spatial resolution limits its ability to detect active fires and the determination of effective fire temperature and effective fire area for smaller fires are impossible with MODIS, because their TIR signals are around the same level as the background variation [11]. The Bispectral InfraRed Detection (BIRD) small satellite mission, launched on 22 October 2001, was developed by the German Aerospace Center (DLR), and continued to make observations until the beginning of 2004. The principal BIRD imaging payload includes the Hotspot Recognition System HSRS with channels in the Mid-Infrared (MIR,3.4-4.2 μm) and the Thermal Infrared (TIR 8.5-9.3 μm) spectral ranges and the Wide-Angle Optoelectronic Stereo Scanner WAOSS-B with a nadir channel in Near-Infrared (NIR:0.84-0.90 μm). The ground resolution of the BIRD nadir channels is 185 m in the NIR and 370 m in the MIR and TIR. However, all three channels have the same sampling step of 185 m due to a factor of 2 oversampling of the MIR and TIR data. BIRD can detect hot targets as small as 1/7 of a MODIS or AVHRR pixel [12-13].

The existing fire detection algorithms can be classified as two categories: fixed-threshold algorithms and contextual algorithms [14-17]. A fixed-threshold algorithm will be set before judging whether the pixel is a fire one or not in the algorithm. The image is processed pixel by pixel by this method. On the contrary, the contextual algorithm is more flexible in the judgment of a fire pixel. First, it distinguishes the potential fire pixels based on a fixed threshold. Second, the statistics from ambient pixels are computed. Finally, the fire pixels from potential fire pixels are confirmed based on the ambient pixel statistics. Many contextual fire detection algorithms have been developed for the AVHRR, MODIS, ATSR, GOES and VIRS sensors [18-25]. The MODIS fire algorithm has been developing and increasing in maturity. In the previous version it had some disadvantages [19, 22, 26], such as the “hole” which was found in cases where the brightness temperatures of neighboring non-fire pixels were high enough to cause a centre pixel containing a hot and/or large fire not to be detected. Meanwhile, false detections may occur over some deserts and bare soils. The detection algorithm was improved greatly in the MODIS Fire Product version 4 [10]. Furthermore, in this version, the MODIS algorithm has considered the effect of coastlines, and includes a formula for the sun glint [10]. Based on the advantages of the MODIS algorithm, HJ-1B data is expected to be more sensitive to smaller and cooler fires than MODIS or AVHRR due to the finer spatial resolution. Further, AVHRR/MODIS fire detection algorithms may not be appropriate because of the channel differences. Modifications or new algorithms are necessary.

To demonstrate the potential ability of active fire detection and seek a suitable algorithm for the new high spatial resolution infrared sensor, simulated HJ-1B images were used in this paper. After a brief description of the HJ-1B sensor payloads, the simulation method of HJ-1B data which include RED, NIR, MIR and TIR channels is introduced. Fires of various sizes and temperatures in a wide range of terrestrial biomes and climates were generated in the simulated images. The MODTRAN 4 atmospheric model was used to model various atmospheric conditions. Considering the characteristics of HJ-1B sensor, which is much sensitive to smaller and cooler fires due to its fine spatial resolution, a contextual fire detection algorithm was proposed and its performance was tested using the simulated HJ-1B images. Probabilities of successful fire detection, commission error and omission error were quantified as functions of fire temperature and fire area in various atmospheric conditions.

## 2. HJ-1B instrument description

China plans to launch two small optical satellites and one small SAR satellite, called the “2+1” Project, in 2008. The project is also called as the Small Satellite Constellation for Disaster Prediction and Monitoring which will be participated in Disaster Monitoring Constellation (DMC). The establishment of a small satellite constellation will make China able to monitor disasters and environmental changes more efficiently. The revisit time of the satellite constellation is 48-96 hours. The satellite designed for fire detection is also called a HJ-1B satellite, and carries two CCD cameras and one thermal scanner. The HJ-1B satellite payload consists of two wide field multi-spectral cameras and one Infrared scanner, whose main specifications are shown in Table 1.

The HJ-1B satellite has been specifically designed to support detection and quantitative characterization of various disasters, such as active fires, active volcanoes, earthquake or others. The Infrared camera possesses mid-infrared (MIR: 3.50∼3.90 μm) and thermal infrared (TIR: 10.5∼12.5μm) spectral channels with 150 m and 300 m resolutions, respectively. The Multi-spectral CCD camera possesses RED (0.63∼0.69 μm) and NIR (0.76∼0.90 μm) spectral channels with a 30 m resolution. The revisit interval of Multi-spectral CCD camera and infrared scanner is 48 hours.

Compared with the MODIS MIR (3.9 μm) band, the MIR channel of HJ-1B is more sensitive to fires due to the shorter wavelength (3.50–3.90 μm) used, but on the other hand, it is more affected by sunlight. HJ-1B has higher spatial resolutions of 300 m and 150 m for the TIR and MIR channels, respectively. Such high resolutions are useful for the accurate estimation of the background temperature of ambient pixels, which is important for the bi-spectral method [26]. The HJ-1B sensor has a NEdT of 1 K. Its saturation levels are 500 K and 340 K for MIR and TIR channels, respectively.

## 3. HJ-1B images simulation

### 3.1. Airborne data

The data used to simulate HJ-1B data in this paper was obtained from thermal infrared (TIR) images acquired by an airborne hyperspectral scanner (AHS). The AHS instrument has 80 spectral bands covering the visible and near infrared (VNIR), short wave infrared (SWIR), mid-infrared (MIR) and thermal infrared (TIR) spectral range. The instrument is operated by the Spanish Instituto Nacional de Técnica Aerospacial (INTA) and it has been involved in several field campaigns since 2004 [27]. The simulated HJ-1B image in this paper was obtained from the fusion of the land surface temperature images from the 10 AHS thermal infrared bands, from 71 to 80, located in the region between 8 and 13 μm with a spatial resolution of 2.5 m at nadir. The study area was located at Cabauw, Loobos and Speulderbos in The Netherlands [40]. The dates of the airborne-AHS data mainly included the 15^th^ and 18^th^ of July 2004 and 13^th^ of June 2006. Grasslands, forests, croplands, bare soil, buildings, roads, rivers, etc. were covered in these areas. The grassland is mainly distributed in Cabauw and the forest mainly distributed in Loobos and Speulderbos [40].

### 3.2. Fire characteristics

In general, the temperatures of active fires vary from 800 K to 1,200 K, and that of smoldering fires range from 600 K to 800 K [28]. Extremely large fires with temperatures higher than 1,400 K may occur in forested areas, but with a low probability, and consequently, they are not considered in the general research. Fire areas from 10 m^2^ to a maximum of 10,000 m^2^ were simulated in the study, corresponding to the fractions from 0.01% to 10% of a 300 m resolution of HJ-1B pixel. Fire emissivities were assumed to be an average value of 0.95 in both MIR and TIR channels.

### 3.3. Background characteristics

Ambient pixels in a square window centered on the candidate fire pixel are used to estimate the mean non-fire “background” values of the MIR and TIR channels. By having a higher spatial resolution, the spatial variance of HJ-1B data is also larger than that of MODIS data, which will lead to the remarkable temperature discrepancy between pixels. Thus, the selection of candidate pixels which may have the same background temperature as the fire pixel is more difficult.

### 3.4. View geometries and atmospheric conditions

The scan angle of HJ-1B may reach a maximum of 29° from the nadir, and the solar zenith angles were assumed to change from 0° to 60°. The latter is important in MIR channel data simulation because solar zenith angle can affect the performance of the algorithm. The satellite altitude was fixed to 650 km. Six standard atmospheric conditions, which include tropical, temperate, mid-latitude and sub-Arctic climates, together with 23 km visibilities, were selected in the MODTRAN 4 based simulation. A brief description of MODTRAN 4 is given in the next section.

### 3.5. A brief introduction to MODTRAN

MODTRAN (MODerate spectral resolution atmospheric TRANsmittance and radiance code) was developed by the U.S. AFRL/VSBT (Air Force Research Lab, Space Vehicles Directorate) in collaboration with Spectral Sciences, Inc. MODTRAN code calculates atmospheric transmittance and radiance for frequencies from 0 to 50,000 cm^-1^ at moderate spectral resolution, primarily 2 cm^-1^ (20 cm^-1^ in the UV) [31]. The original development of MODTRAN was driven by a need for higher spectral resolution and greater accuracy than that provided by the LOWTRAN series of band model algorithms. Except for its molecular band model parameterization, MODTRAN adopts all the (now fully obsolete) LOWTRAN 7 capabilities, including spherical refractive geometry, solar and lunar source functions, scattering (Rayleigh, Mie, single and multiple), and default profiles (gases, aerosols, clouds, fogs and rain). MODTRAN is useful both in sensor signal simulation and atmospheric correction [31, 32]. The latest version, MODTRAN 4.0, was used in this research for the land surface temperature and emissivity retrieval and HJ-1B image simulation. Six standard atmospheric conditions were assumed in the simulation. The key variables of these six conditions are listed in Table 3.

### 3.6. Simulation of HJ-1B images

The RED and NIR channel of HJ-1B sensor were denoted as channels 1 and 2, which correspond to the B3 and B4 bands of the CCD Camera, respectively. The MIR and TIR channel were denoted as channels 3 and 4, which correspond to the B3 and B4 bands of the Infrared Scanner, respectively. The VNIR, MIR and TIR channel of HJ-1B sensor have 30 m, 150 m and 300 m resolution, respectively, and the VNIR and MIR channel data should be aggregated to the TIR channel's resolution. Because only the daytime AHS data was available, both the simulation and fire detection algorithm were focused on the daytime situation.

**Figure 1.**
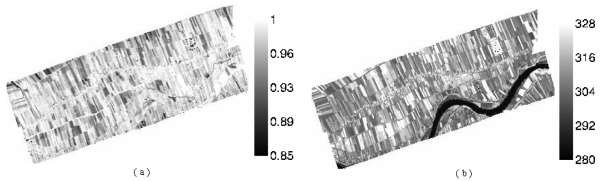
Retrieval of land surface emissivity and temperature (K) of HJ-1B from simulated AHS data, (a) land surface emissivity, (b) land surface temperature.

First, the real-time atmospheric profile was used to correct the atmospheric effect of AHS data based on MODTRAN 4 [34]. The surface temperature and emissivity of an AHS pixel were obtaned by the TES algorithm [35]. Secondly, the surface temperature and emissivity at the scale of a HJ-1B pixel were got by spectrum transform [36] and spatial aggregation at a 300 m scale, which is the resolution of HJ-1B's thermal channel [37]. The results are shown in Figure 1.

Thirdly, fires with varied temperature and area were added randomly into the HJ-1B pixels. In order to enable the fire simulation to be much more rational, the fire was added only to the vegetation covered pixels. Thus, fire was not simulated for bare soil, water surfaces and clouds. Two hundred fire pixels were generated by the following steps:

1. The pixels were selected randomly in the image to add simulated fire.
2. Cloud pixels were removed by the limitation of *T*_4_ > 265 K.
3. Vegetation covered pixels were selected by the condition of NDV ≥ 0.3.
4. Fire temperatures were assumed to be from 600 K to 1,200 K.
5. Fire fractions were assumed to be from 0.0001 to 0.1.

Finally, the apparent radiances of the MIR and TIR data of HJ-1B were generated by equations (1) and (2). MODTRAN 4 was used to obtain the atmospheric parameters in these two equations under various solar and atmospheric conditions which may provide a statistical evaluation of fire detection algorithm proposed in the next section. Standard atmospheric conditions which include Tropical, Mid-Latitude Summer, Mid-Latitude Winter and US Standard Atmosphere were adopted in the simulation.

(1) $$L(T_{3})=\tau_{3}\times {ɛ}_{3}\times p\times B(T_{f},\lambda_{3})+(1-p)\times L(T_{3b})+p\times \mathrm{Latm},3$$

(2) $$L(T_{4})=\tau_{4}\times {ɛ}_{4}\times p\times B(T_{f},\lambda_{4})+(1-p)\times L(T_{4b})+p\times \mathrm{Latm},4$$

where, *L*(*T*_3_) and *L*(*T*_4_) are the TOA radiances of HJ-1B MIR and TIR channels respectively; *τ*_3_ and *τ*_4_ are the atmospheric transmittances calculated by MODTRAN 4; *ε*_3_ and *ε*_4_ are channel emissivities of fire; *p* is the fraction of fire area; *T_f_* is fire temperature simulated; *λ* is the center wavelength of each channel; *B*(*T*, *λ*) is the Planck function; *L*(*T*_3_*_b_*) and *L*(*T*_4_*_b_*) are background radiance; *Latm*,3 and *Latm*,4 are the upwelling atmospheric radiance obtained from MODTRAN 4. Because *p* and *Latm*,*i* (*i*=3, 4) are both small, the quantities of *p* × *Latm*,3 and *p* × *Latm*,4 are usually omitted [11]. The sensitivity analysis of each parameter can be found in literature [38].

## 4. Fire detection algorithms

**Taking** into account that HJ-1B was used to detect fire in various atmospheric conditions, the HJ-1B fire detection algorithm was designed to be an adaptive, contextual algorithm rather than a fixed threshold method.

Brightness temperatures derived from channel 3 (MIR channel) and channel 4 (TIR channel) of the thermal sensor onboard HJ-1B, which are denoted as *T*_3_ and *T*_4_, are used in the algorithm. Channel 4 is also used for cloud screening as the temperature of cloud appears rather lower in this channel than others. The data of red and near-infrared channels are aggregated to 300 m for false alarm rejection and cloud masking. The reflectance of these two channels is denoted as *ρ*_1_ and *ρ*_2_, respectively.

### 4.1. Cloud and fire scars screening

The reasonable selection of background pixels is the key to estimating the adaptive thresholds used in the contextual algorithm. If the background pixels selected are unrepresentative of the wider terrestrial background conditions, it may lead to significant offsets in the estimated background characteristics, and this will result in fire detection omission errors or commission errors. Thus, the contaminated pixels must be rejected before estimating the background characteristics and detecting fires. These noises mainly include cloud, water, sun glint, fire scars, etc.

Daytime cloud contamination of MIR channel was the most common source of false detections. Being illuminated by sunlight, clouds can elevate the TOA MIR radiance due to the reflected sunlight and reduce the TOA TIR radiance due to their low temperatures [28]. Cloud detection was performed using a technique based on that used in the production of the IGBP AVHRR-derived Global Fire Product [29]. But due to the different spectral response for cloud, the thresholds used to cloud detection have been modified empirically for HJ-1B. If the following condition is satisfied, the pixel will be considered to be cloud-obscured:

(3) $$\begin{array}{l} \rho_{1}+\rho_{2}>0.8\;or\;T_{4}<265K\;\mathrm{or}\\ \rho_{1}+\rho_{2}>0.6\;\mathrm{and}\;T_{4}<285K \end{array}$$

Water detection is mainly based on the fact of that the reflectance of water in red and NIR channels are very low, and NDVI is less than zero [10]. A pixel is assumed to be water under the following conditions:

(4) $$\rho_{1}<0.1\;\mathrm{and}\;\rho_{2}<0.1\mathrm{and}\text{NDVI}<0$$

Fire scars usually show higher temperatures than the background. They may cause overestimation of background temperatures. The NIR data provides very effective discrimination of post-fire surfaces (burn scars) since the reflectance difference between unburned and burned pixels is often maximized in this spectral region. So the NIR reflectance (due to lower reflectance of fire scar) can be used to eliminate the effect of fire scars. One pixel was contaminated by fire scars if it meets the condition given by: *ρ*_2_ < 0.2 [13].

### 4.2. Sun glint screening

Sun glint usually occurs at surfaces which may cause mirror reflectance, such as small bodies of water, wet soils and cirrus clouds. It may cause false alarms [10]. As a result, such surfaces should also be eliminated. The pixels contaminated by sun glint should not be considered when calculating background statistical information. The absolute difference of RED reflectance and NIR reflectance is greater than 0.01 under sun glint conditions. Sun glint can also be rejected with a scheme proposed by Giglio *et al.*, which uses the angle between vectors pointing in the surface-to-satellite and specular reflection direction [10], which can be expressed as the function of the relative azimuth angle, view and solar zenith angle. The simple absolute difference method was used in this paper to do sun glint screening.

### 4.3. Absolute threshold based identification

A preliminary classification is used to eliminate obvious non-fire pixels. Those pixels will not be considered in the following tests. To detect large fires, a simple absolute threshold test is performed for every non-obscured vegetation pixel throughout the whole image. This threshold should be set as sufficiently high as possible to identify the very unambiguous fire pixels, i.e. those have little chance of being a false alarm.

The absolute threshold criterion remains the same with that proposed in the original algorithm [19], i.e., *T*_3_ > 360 K. This is necessary because this threshold can solve the known “hole” problem, which had been described in detail in the literature [19]. In despite of the higher threshold, an adequate sun glint removal is still necessary to reduce false alarm probabilities.

### 4.4. Identification of potential fire pixels

The identification of the potential fire pixels is based on the fixed threshold algorithm. A pixel is confirmed as a potential fire pixel if *T*_3_ > 308 K, *T*_34_ > 8 K, *ρ*_2_ < 0.3, where *T*_34_ = *T*_3_ – *T*_4_, otherwise it is classified as non-fire. It is assumed that the brightness temperature of one pixel is larger than 308 K if it includes fire. The difference of brightness temperature between MIR and TIR channels mainly aims at eliminating the land surfaces with high temperatures other than fires, such as bare soil, rock, and road. Meanwhile, it is assumed that the reflectance of NIR channel is very low when fire has occurred [23]. These relatively low thresholds are expected to improve the likelihood of detecting smaller and/or cooler fires which are frequently missed when more conservative thresholds are used. The threshold of MIR channel is very important for fire detection because the active fires show maximum spectral emission in the MIR spectral range.

**Figure 2.**
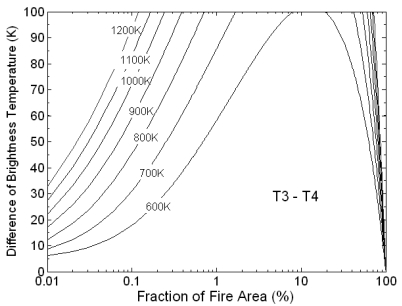
The difference of brightness temperature between MIR and TIR channel of HJ-1B data.

The difference of brightness temperature between MIR and TIR channel has been simulated as the function of fraction of fire area under US Standard Atmosphere condition and 23km visibility. The results are shown in Figure 2. The simulated fraction of fire area ranges from 0.01% to 100% and the temperature ranges from 600 K to 1,200 K. It is clear that the difference of brightness temperatures tend to be zero when the fire fraction is close to 100%. As a result, the large fires may be omitted. The absolute threshold based test described above is necessary.

### 4.5. Selection of background pixels

If the pixel has been identified as a potential fire pixel, the radiant statistical information of its neighboring pixels will be adopted to estimate the brightness temperatures of the fire-free proportion in it. A valid background pixel should meet the following conditions: (1) it is not covered by clouds, which are identified with an external cloud mask; (2) It is not itself a potential fire pixel; (3) it was not classified as water, sun glint, fire scar or data gap. At first a 5×5 window (1.5×1.5km) centered on the potential fire pixel is selected to collect the background information. If the valid background pixels in this window are less than 25%, it can be increased grow up to 30×30 pixels (9×9km) until 25% valid pixels are found and the number of valid background pixels is at least eight. If a sufficient number of valid background pixels can be identified, several statistical values will be computed for fire detection; otherwise the pixel is classified as an “unknown” pixel which will be not analyzed in the following tests [10, 18]. These computed statistics mainly include:

*T̅*_3_*_B_*, the mean of *T*_3_ for the valid neighboring pixels
*δ*_3_*_B_*, the mean absolute deviation of *T*_3_ for the valid neighboring pixels
*T̅*_34_*_B_*, the mean of *T*_3_ – *T*_4_ for the valid neighboring pixels
*δ*_34_*_B_*, the mean absolute deviation of *T*_3_ – *T*_4_ for the valid neighboring pixels

The mean absolute deviation is employed as a measure of statistic properties of the background window rather than the standard deviation since it is more efficient to the normal and some non-normal distributions [30].

### 4.6. Identification of fire pixels

In summary, a pixel will be recognized as a fire pixel after the following steps:

1. It is thought as a potential hot pixel if *T*_3_ > 308 K, *T*_34_ > 8 K, and *ρ*_2_ < 0.3;
2. A pixel is assumed to be cloud if *ρ*_1_ + *ρ*_2_ > 0.8 *or T*_4_ < 265 K or *ρ*_1_ + *ρ*_2_ > 0.6 *and T*_4_ < 285 K
3. A pixel is assumed to be water if *ρ*_1_ < 0.1 *and ρ*_2_ < 0.1 *and* NDVI < 0
4. It is recognized as strong sun glints if |*ρ*_1_ – *ρ*_2_| < 0.01. [6]
5. It is recognized as the highly reflective clouds if *ρ*_2_ > 0.6;
6. If one of the following tests (equation (5) or (6)) can be satisfied, it will be classified as a fire pixel:

(5) $$T_{3}>360K$$

(6) $$\{\begin{array}{c} T_{3}>\bar{T_{3B}}+3.5\times \delta_{3B}\\ T_{4}>\bar{T_{4B}}+\delta_{4B}-4K\\ T_{34}>\mathrm{MAX}(\bar{T_{34B}}+\delta_{34B},8K) \end{array}$$

Equation (5) has been described in 4.3. The first expression of equation (6) means that if the brightness temperature of MIR channel is higher enough, compared with the mean background brightness temperature of the same channel, the pixel may be a fire pixel.

**Figure 3.**
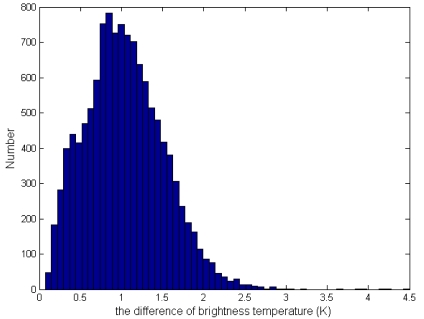
An illustration of the histogram of *δ*_34_*_B_*

**Figure 4.**
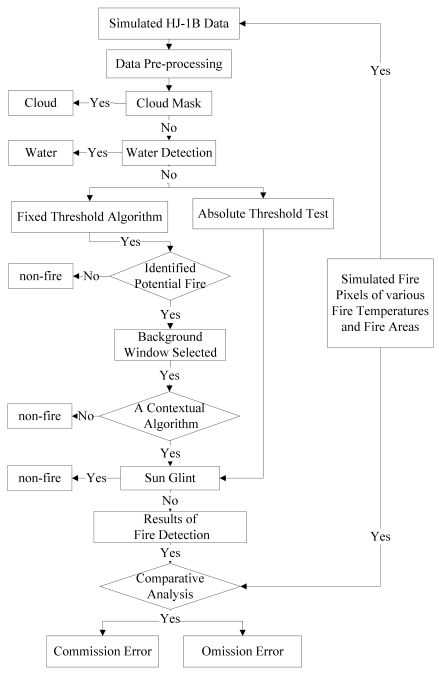
The flow chart of fire detection.

The second expression of equation (6), which is restricted to daytime pixels, is primarily used to reject small convective cloud pixels that can appear warm in the MIR channel (due to reflected sunlight) yet cool in the TIR channel [10, 28]. It can also help reduce coastal false alarms that sometimes occur when cooler water pixels are unknowingly included in the background window [10]. The last expression of equation (6) is that the difference of brightness temperature of MIR and TIR channel should be larger enough. The histogram of *δ*_34_*_B_*, shown in Figure 3, indicates that the mean absolute deviation is far less than 8 K. The detailed flow chart of fire detection is shown in Figure 4:

## 5. Results and analysis

The performance of the fire detection algorithm can be evaluated using of the probabilities of successful detection and the probabilities of false alarms (i.e. commission error). It is evident that the higher probability of fire detection and the lower probability of false alarms, the better performance of the algorithm.

**Figure 5.**
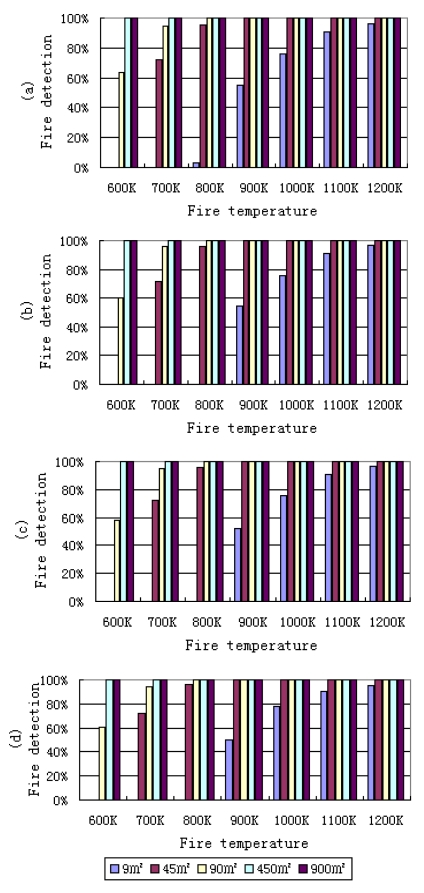
Probabilities of fire detection in nadir view for different fire area (m^2^) and temperature (K) under US standard atmospheric conditions with a visibility of 23 km. The solar zenith angles are 0, 30, 45 and 60 degree for (a), (b), (c) and (d), respectively.

Here the probability of fire detection is defined as the ratio between the true fire pixels which can be detected by the algorithm and the total number of fire pixels simulated. Four different solar zenith angles, 0°, 30°, 45° and 60° were considered under US Standard Atmospheric conditions in the simulation (Figure 5). It can be found that the probability of fire detection will be greatly affected by fire temperature and fire area. Solar zenith angle can also slightly affect the performance of algorithm if the background temperature had not changed. In fact, if we consider the diurnal variation of temperature, a fire may be easier to be detected in nighttime or when the solar elevation is low. This is because the temperature difference between a fire pixel and its neighborhood in MIR channel is relative large. It is clear from the simulation results that the contextual algorithm exhibits a good performance for the larger/hotter fires detection under standard atmospheric conditions. It also can be seen from Figure 5 that when the simulated fire area is larger than 45 m^2^ and the simulated fire temperature is larger than 800 K, the algorithm has a higher probability of detection. But if the fire area is smaller than 10 m^2^, only when the fire temperature is larger than 900 K may the fire be detected.

**Figure 6.**
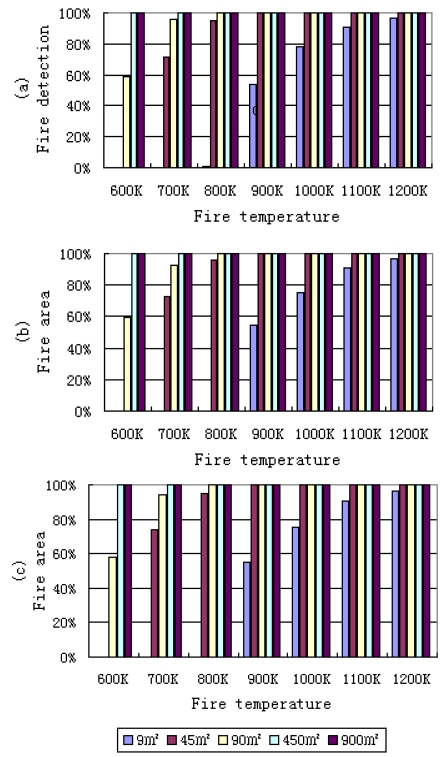
Probabilities of fire detection in nadir view for different fire areas (m^2^) and temperatures (K) under standard atmospheric conditions with a visibility of 23 km; (a) tropical; (b) midwinter; (c) midsummer.

The probabilities of fire detection in nadir view for different fire areas and temperatures under three standard atmospheric conditions are shown in Figure 6. It can be found that different atmospheric conditions can also slightly affect the results of fire detection only when the fire temperature and fire area are small. As in Figure 5, from Figure 6 it is also evident that with the increase of the fire area and fire temperature, the probability of fire detection increases quickly. When a fire area is smaller than 9 m^2^ and fire temperature is less than 800 K, the probability of detection is zero, i.e. fires cannot be detected under these conditions. But with an increasing in fire area, such as a 45 m^2^ fire area and about 800 K temperature, the probability of fire detection is about 95%.

**Figure 7.**
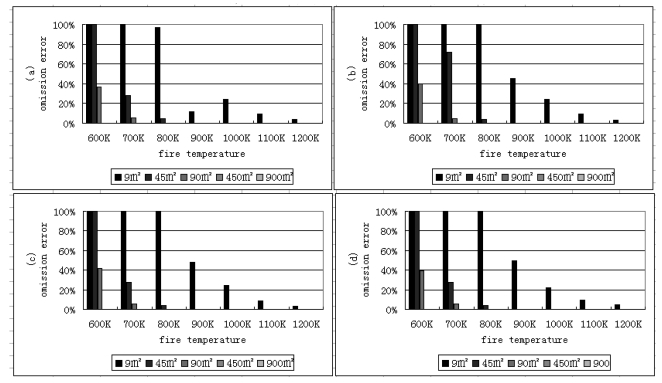
Omission errors of fire detection for different fire areas (m^2^) and temperatures (K) under US standard atmospheric conditions with a visibility of 23 km. The solar zenith angles are 0, 30, 45 and 60 degree for (a), (b), (c) and (d), respectively.

The omission pixels are defined as the pixels including fire, but which cannot be detected using the algorithm. The omission error is equal to the difference between one hundred percent and probability of fire detection [28]. The results of omission errors shown in Figure 7 are the difference between 100 percent and the detection probability shown in Figure 5. Omission errors will occur when the fire area and fire temperature are small. This may also happen when many fire pixels assemble and form a “cluster”. Because the temperatures of all neighboring pixels are high and homogeneous in this “cluster”, a pixel with a lower temperature can be misidentified as a non-fire pixel. Another reason is the high thresholds. Higher thresholds can lead the higher omission errors, while lower thresholds can cause the higher commission errors.

**Figure 8.**
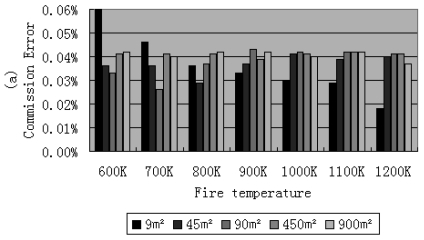

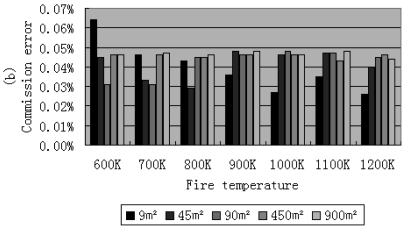
Commission errors of fire detection for different fire areas (m^2^) and temperatures (K) Under the US standard atmospheric conditions with a visibility of 23 km. The solar zenith angles are 0 and 30 degree for (a) and (b), respectively

The commission pixels are defined as those, not including fire, but which have been recognized as fire. The commission error is defined as the ratio between the number of non-fire pixels detected as fire pixels and the sum of fire pixels [28]. The commission errors of fire detection for different fire areas and temperatures under the US standard atmospheric condition with a visibility of 23 km are shown in Figure 8.

It was found that the thresholds can affect the results of the commission and omission errors. The commission error can be reduced by a higher threshold, but the omission error can be reduced by a lower threshold. In this framework, higher thresholds were used to get small commission errors but caused more omission errors especially when both of the fire temperature and the fire area were small. But when fire area is larger than 45 m^2^ and fire temperature is larger than 800 K, the omission error is very small. By using the thresholds listed in this paper, the commission error was controlled lower than 0.1%.

## 6. Conclusions

**The** fire detection ability of HJ-1B sensors was evaluated based on a simulation. A contextual daytime fire detection algorithm for HJ-1B data was proposed. The work presented in this paper provides both qualitative and quantitative evaluation of simulated HJ-1B fire detection with its characteristics. The analysis has shown the limitations and advantages of HJ-1B data for this purpose. It can be seen from the simulated results that the algorithm performance varies under different fire, atmospheric conditions, solar zenith angle, etc. The performance has been characterized in terms of probabilities of fire detection and false alarm as functions of fire temperature and fire area. There are several general implications from this work: (i) HJ-1B data is capable of detecting the fires as small as about 10 m^2^. This advantage is due to its fine spatial resolution compared with MODIS or AVHRR data. However, the fire temperature must be enough high to detect fires with this algorithm when the fire area is about 10 m^2^, otherwise, omission errors may occur; (ii) solar zenith angle and different atmospheric conditions can slightly affect the performance of the algorithm; (iii) the factors affecting the performance mainly include the threshold, land surface situation, fire temperature and fire area, etc. High thresholds can reduce commission errors, but can cause large omission errors, and *vice versa*. A nighttime fire detection algorithm was not proposed in this work because only daytime AHS data are available. The algorithm proposed in this paper is more similar to a regional fire detection algorithm, because the study area is not large enough. The simulation also provides an important method for fire detection algorithm of HJ-1B evaluation, but which can not represent completely the real landscape. So this work is expected to be tested using the provided a candidate fire detection algorithm for the HJ-1B satellite in the next study.

## Figures and Tables

**Table 1.** Specifications of the HJ-1B main payloads.

**Sensor**	**CCD Camera**				**Infrared Scanner**			
**Parameter**								
**Band number**	Four bands				Four bands			
**Spectral bands (μm)**	B1	B2	B3	B4	B1	B2	B3	B4
	0.43∼0.52	0.52∼0.60	0.63∼0.69	0.76∼0.90	0.75∼1.10	1.55∼1.75	3.50∼3.90	10.5∼12.5
**MTF**	≥0.20	≥0.20	≥0.20	≥0.14	0.28	0.27	0.26	0.25
**Radiant Resolution (NEdρ or NEdT)**	\				0.5%	0.5%	≤1K (500K)	≤1K (340K)
**Ground Resolution at nadir**	30m				300m(10.5∼12.5μm,TIR), 150m(others)			
**Swath width**	360km*2 swath				720km			
**Field of view**	31°				±29°			
**S/N**	average≥48dB, min>6dB				\			
**Quantization**	8 bit				10 bit			
**Detector type**	CCD				infrared scanner			
**Revisit time**	48 hours				48 hours			
**Orbit**	sun-synchronous				sun-synchronous			

**Table 2.** The details of AHS Thermal bands from N0.71 to N0.80 [39].

**Band Number**	**Spectral range (μm)**	**Center wavelength (μm)**
71	7.90-8.37	8.13
72	8.43-8.84	8.63
73	8.93-9.33	9.13
74	9.36-9.79	9.58
75	9.85-10.26	10.06
76	10.29-10.83	10.57
77	10.90-11.43	11.17
78	11.49-12.01	11.74
79	12.09-12.60	12.36
80	12.67-13.20	12.94

**Table 3.** Air temperatures at the first boundary and the total water vapor contents of the six standard model atmospheres prescribed in MODTRAN [33].

**Model atmosphere**	***T*_0_ (K)**	**W (g/cm^2^)**
Tropical	299.7	4.11
Mid-Latitude Summer	294.2	2.92
Mid-Latitude Winter	272.2	0.85
Sub-Arctic Summer	287.2	2.08
Sub-Arctic Winter	257.2	0.42
1976 US Standard	288.3	1.42
